# Multispecialty Management of Pancreaticopleural Fistula With Recurrent Pleural Effusions: A Case Report and Literature Review

**DOI:** 10.7759/cureus.101458

**Published:** 2026-01-13

**Authors:** Navin Prasad, Rani Bijjam, Pradeep R Kathi, Vipin Mittal

**Affiliations:** 1 Internal Medicine, Presbyterian Hospital, Albuquerque, USA; 2 Gastroenterology and Hepatology, Presbyterian Hospital, Albuquerque, USA

**Keywords:** endoscopy, ercp, pancreaticopleural fistula, pancreatitis, pseudocyst

## Abstract

Pancreaticopleural fistula (PPF) is an uncommon complication of pancreatitis that typically presents with respiratory symptoms rather than classic epigastric pain. PPF often occurs in settings of alcoholic pancreatitis with pancreatic pseudocyst rupture or ductal disruption, leading to abnormal pancreas-pleural communication.

A 50-year-old woman with alcohol and tobacco use disorders presented with progressive dyspnea. She was found to have a left pleural effusion and acute pancreatitis. She experienced recurrent admissions for persistent pleural effusions despite appropriate chest tube drainage. Pleural fluid revealed markedly elevated lipase, and serial imaging identified a pseudocyst and PPF. A disconnected pancreatic duct was endoscopically stented. Her course included management with surgical decortication, talc pleurodesis, and multiple chest tubes, as well as complications of an enlarging pancreatic pseudocyst, splenic vein thrombosis, and pleural empyema. She was declined as a surgical candidate for pancreatectomy due to high operative risk. A multidisciplinary team recommended conservative management with parenteral nutrition and fistula suppression with octreotide.

This case underscores the importance of early suspicion for PPF in patients with pancreatitis and recurrent pleural effusions, emphasizing the role of pleural enzyme testing, targeted imaging, and coordinated multispecialty management to reduce diagnostic delays and guide timely intervention.

## Introduction

Pancreaticopleural fistula (PPF) is an uncommon complication of pancreatitis, with a reported incidence of around 0.4% in those with pancreatic disease [1]. PPF usually occurs in cases involving chronic alcohol use, pseudocyst formation, and disruption of the pancreatic duct leading to left-sided pleural effusion [1-2]. Unlike in classic uncomplicated pancreatitis, which presents with abdominal pain, the diagnosis of PPF can be delayed as patients frequently present with respiratory symptoms such as dyspnea or pleurisy [1-3].

Due to its atypical presentation, diagnosing PPF requires a high index of suspicion. Pleural fluid analysis typically reveals markedly elevated levels of amylase and/or lipase, with lipase offering greater sensitivity and specificity [3]. Abdominal imaging with CT or magnetic resonance cholangiopancreatography (MRCP) identifies pseudocysts, peripancreatic collections, and fistulous tracts, although small or subtle tracts may be easily overlooked [4]. Management comprises drainage of pleural collections, endoscopic stenting of pancreatic ductal defects, and consideration of surgery for fistula intervention [1-8].

Here, we present the case of a woman with alcoholic pancreatitis with recurrent pleural effusions in the setting of PPF and disconnected duct syndrome. Her course illustrates the diagnostic challenges, complex procedural decision-making, and limitations in management that are inherent to treating this condition.

## Case presentation

A 50-year-old woman with alcohol use disorder for over 20 years, tobacco use with over 40 pack-year smoking history, and post-traumatic stress disorder (PTSD) presented to our hospital with progressive dyspnea (Table 1). She had no prior history of pancreatitis.

**Table 1:** Clinical timeline of recurrent hospitalizations, diagnostic findings, and management in a patient with pancreaticopleural fistula. CT, computed tomography; A/P, abdomen and pelvis; VATS, video-assisted thoracoscopic surgery; ERCP, endoscopic retrograde cholangiopancreatography

Admission	Presentation	Imaging findings	Pleural fluid analysis	Management
First	Dyspnea	CT chest: large effusion; CT A/P: normal pancreas	Exudative, hemorrhagic (amylase/lipase not sent)	Chest tube
Second	Pleuritic chest pain, epigastric pain	CT: peripancreatic fluid, walled-off necrosis	Exudative; pleural amylase not measurable	Chest tube and VATS with decortication
Third	Recurrent abdominal pain, pleuritic chest pain, foul-smelling chest tube site drainage	CT: loculated hydropneumothorax, persistent peripancreatic pseudocyst with connection to pleural space	Lipase ↑; amylase undetectable	Chest tube and talc pleurodesis; ERCP stenting of disconnected duct; subspecialty surgery declined distal pancreatectomy; conservative therapy including IV nutrition

First two hospitalizations

**Figure 1:**
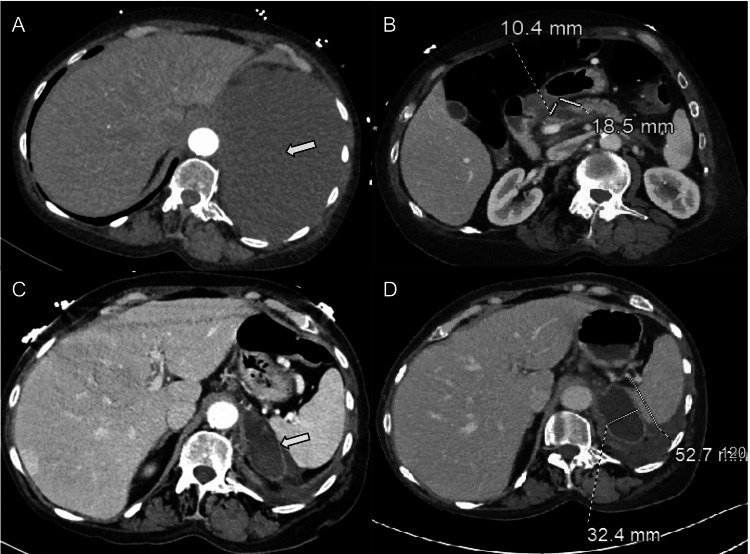
Chronological axial contrast-enhanced CT imaging demonstrating progression of a pancreaticopleural fistula. (A) Initial chest CT demonstrating a large left-sided pleural effusion (arrow) at first admission. (B) Abdominal CT demonstrating a pancreatic neck pseudocyst with surrounding inflammatory changes consistent with acute pancreatitis at first admission. (C) Abdominal CT demonstrating a pancreatic tail pseudocyst with fistulous communication into the left pleural space (arrow) during the third admission. (D) Follow-up CT demonstrating interval enlargement of the pancreatic tail pseudocyst during the third admission.

On initial presentation, chest CTA demonstrated a large left-sided pleural effusion, while abdominal CT showed no pancreatic abnormalities (Figure 1A). Thoracentesis revealed an exudative, hemorrhagic effusion, and chest tube placement led to rapid symptomatic improvement. Interval imaging demonstrated evolving peripancreatic inflammation with a 1.9-cm cystic lesion at the pancreatic neck (Figure 1B), accompanied by elevated serum amylase (315 U/L) and lipase (416 U/L), consistent with acute pancreatitis.

The patient was readmitted one week later with recurrent epigastric and pleuritic chest pain, markedly elevated serum lipase (1,946 U/L), and persistent peripancreatic fluid with stable walled-off necrosis on CT. Repeat pleural drainage demonstrated an exudative effusion, and she underwent video-assisted thoracoscopic surgery (VATS) with decortication, resulting in clinical improvement and chest tube removal.

Third hospitalization

The patient was readmitted several days later with recurrent abdominal and pleuritic chest pain and foul-smelling drainage from a prior chest tube site. CT imaging demonstrated a loculated hydropneumothorax with a moderate left pleural effusion and a persistent pancreatic pseudocyst. Pleural fluid analysis revealed markedly elevated lipase (>3,000 U/L) with undetectable amylase, strongly suggesting pancreaticopleural fistula.

Despite pleural drainage and bedside talc pleurodesis, serial imaging demonstrated a dominant pancreatic tail pseudocyst extending through the left hemidiaphragm into the pleural space (Figure 1C). Endoscopic retrograde cholangiopancreatography (ERCP) subsequently confirmed a disconnected pancreatic duct at the neck-body junction with contrast extravasation; attempts at ductal bridging were unsuccessful, and a transpapillary pancreatic duct stent was placed for pressure diversion.

The patient’s course was complicated by persistent fluid collections, interval enlargement of the pancreatic pseudocyst, acute splenic vein thrombosis, and left-sided empyema requiring antibiotics and additional pleural drainage (Figure 1D). Endoscopic cystogastrostomy was deferred because of immature collections and perigastric varices, and surgical resection was considered prohibitively high risk due to extensive fibrosis, vascular thrombosis, and bleeding risk. She improved after conservative management with bowel rest, parenteral nutrition, and intravenous octreotide before discharge to a skilled nursing facility.

## Discussion

PPF is a complication of pancreatitis caused by a ruptured pancreatic duct or pseudocyst extending into the pleural space with leakage of pancreatic enzymes into the pleural cavity [6-7]. PPF is most frequently seen in males (~80%) with alcohol use and resulting chronic pancreatitis. Patients usually present with respiratory symptoms, such as dyspnea, pleuritic pain, or large recurrent effusions [6-8].

A high pleural fluid lipase or amylase level is considered diagnostic of an effusion caused by pancreatitis [3]. However, measurement can be limited by assay interference or lab reporting thresholds, as seen in this case. Imaging with CT and MRCP is critical for identifying pseudocysts, peripancreatic collections, and possible fistulous tracts, though small or partially obstructed fistulas may be missed [4]. MRCP and ERCP are more sensitive for detecting fistulous tracts and evaluating ductal anatomy compared to CT, while ERCP is both diagnostic and therapeutic [4]. MRCP did not detect a fistula in our patient, but serial CT imaging eventually confirmed the diagnosis.

Management of PPF requires drainage of pleural collections and definitive therapy targeting the underlying pancreatic duct disruption; surgically addressing the fistula may also be required if these fail [1-2,6,8]. Endoscopic therapy, primarily with pancreatic duct stenting via ERCP, is preferred as it achieves closure in up to 79% of cases [1,6,8]. In cases with ductal disconnect, endoscopic outcomes may be less favorable as bridging the disrupted segment can be technically challenging [2,8]. Our patient underwent endoscopic stenting, yet persistent collections and empyema complicated her course. Surgical management, such as with distal pancreatectomy were deemed unsafe given her retroperitoneal fibrosis, splenic vein thrombosis, and bleeding risk.

This case highlights the limitations of endoscopic and surgical management in patients with complex anatomy and comorbidities. Multispecialty collaboration with gastroenterology, pulmonology, thoracic surgery, radiology, and surgical oncology across multiple hospital systems was crucial in managing our patients’ acute conditions, comorbidities, and disease-related complications. Ultimately, conservative medical therapy with bowel rest, parenteral nutrition, and somatostatin analogs was pursued after procedural options had reached their limits.

## Conclusions

PPF should be suspected in patients with alcoholic pancreatitis and recurrent or unexplained pleural effusions. This case underscores the diagnostic value of pleural lipase testing when amylase is inconclusive and highlights the importance of careful, targeted imaging review to identify subtle fistulous tracts. Management often requires escalation across endoscopic, surgical, and conservative strategies, particularly in the setting of complex ductal anatomy, with treatment goals focused on stabilization rather than definitive cure when procedural options are limited. Early recognition and coordinated multi-specialty care are essential to reducing diagnostic delay, repeated interventions, and associated complications.
